# Social Media Discourse on Breast Cancer Screening Barriers in Japanese and English: Cross-Sectional Infodemiology Study

**DOI:** 10.2196/97772

**Published:** 2026-07-31

**Authors:** Mitsuo Terada, Rie Kawabori Tahara, Yumi Wanifuchi-Endo, Tomoko Asano, Nanae Horisawa, Kazuki Nozawa, Ayaka Isogai, Nari Kureyama, Hikaru Kawahara, Mika Kotani, Yuya Tanaka, Marie Mizumoto, Atsushi Fushimi, Nami Yamashita, Madoka Iwase, Asumi Iesato, Tatsuya Toyama

**Affiliations:** 1 Department of Breast Surgery, Nagoya City University Graduate School of Medical Sciences, 1 Kawasumi, Mizuho-cho, Mizuho-ku, Nagoya, Aichi, 467-8601, Japan, 81 52-851-5511, 81 52-853-8337; 2 General Incorporated Association BC TUBE, Chiyoda-ku, Tokyo, Japan; 3 Department of Advanced Clinical Research and Development, Nagoya City University, Nagoya, Aichi, Japan; 4 Department of Breast and Endocrine Surgery, Jikei University School of Medicine, Minato-ku, Tokyo, Japan; 5 Breast Oncology Center, Japanese Foundation For Cancer Research, Koto-ku, Tokyo, Japan; 6 Department of Breast and Endocrine Surgery, Nagoya University Hospital, Nagoya, Aichi, Japan; 7 NEXT-Ganken Program, Cancer Cell Diversity Project, Japanese Foundation For Cancer Research, Koto-ku, Tokyo, Japan

**Keywords:** breast cancer, mammography, screening, social media, natural language processing, cross-cultural comparison

## Abstract

**Background:**

Despite clinical advances, breast cancer screening adherence remains stagnant in Japan (<50%) compared with the United States (>70%). Understanding distinct cross-cultural barriers is essential; however, traditional methodologies often fail to capture visceral, real-world individual experiences and hidden deterrents to screening.

**Objective:**

This study aims to characterize and compare cross-cultural informatics profiles of barriers to breast cancer screening across Japanese-language and English-language social media discourse.

**Methods:**

We developed an automated natural language processing pipeline on a cloud-based informatics platform to analyze 46,823 screening-related posts (30,027 in Japanese and 16,796 in English) from X (formerly Twitter) collected in 2025, derived from an initial 76,955 posts after noise exclusion. The methodology integrated large language model–assisted sentiment polarity scoring with strict negation-handling, co-occurrence network topology analysis, and advanced distributional visualizations, including raincloud and ridgeline plots. Subgroup comparisons (prescreening vs postscreening and ultrasound with vs without mammography) were evaluated.

**Results:**

Among the 46,823 screening-related posts, overall sentiment distributions showed no practically meaningful cross-cultural divergence (Cohen *d*=0.049); however, domain-specific analyses revealed sharp disparities in barrier prevalence. Within the English-language cohort containing 16,796 posts, discourse exhibited a concentrated, moderate negative sentiment regarding systemic barriers, featuring “Cost” as the primary barrier (n=1239, 7.4%), while “Pain” ranked considerably lower (n=842, 5.0%). In contrast, the Japanese-language cohort containing 30,027 posts was heavily bottlenecked by psychosomatic barriers, governed by a tightly interconnected network of “Pain,” “Fear,” and “Appointment.” “Pain” emerged as the overwhelmingly dominant barrier (n=5788, 19.3%). In the English-language cohort, “Dense” breasts emerged as a prominent clinical topic due to elevated public awareness, distinct from the financial narrative. The Japanese subgroup analyses identified a temporal transition from anticipatory psychological anxiety (“Fear,” 739/3805, 19.4%) and logistical concerns (“Appointment,” 1556/3805, 40.9%) before screening to a strong persistence of the discomfort memory of “Pain” (1160/7419, 15.6%). Furthermore, sentiment scores for mammography were significantly more negative than those for ultrasound alone (*P*<.001, Cohen *d*=0.264), and pain-related descriptors for ultrasound spiked from 5.0% (106/2110, ultrasound alone) to 18.2% (733/4017) when performed concurrently with mammography.

**Conclusions:**

Despite comparable overall emotional equilibrium, a fundamental dichotomy emerged. The English-language discourse predominantly reflects US-specific systemic financial burdens, whereas the Japanese experience is characterized by emotional volatility transitioning from anticipatory anxiety to a tightly interconnected “Pain-Fear-Appointment” network. Physical discomfort of mammography dominates the screening narrative, overshadowing concurrent painless modalities like ultrasound. Improving adherence in Japan requires individualized pain-mitigating compression protocols and optimized clinical workflows to decouple mammographic discomfort from supplemental screening, thereby preventing pain-associated defensive avoidance and reducing logistical hurdles to improve equitable access.

## Introduction

Breast cancer screening effectively reduces disease-specific mortality through early detection; however, significant global disparities in screening uptake persist [1]. While the United States reports screening rates exceeding 70%, Japan’s participation remains stagnant below 50% [2]. Addressing this gap requires a granular understanding of the “real-world” barriers that deter women from screening. Traditional survey-based methodologies, while foundational, are often limited by recall bias and social desirability bias and fail to capture longitudinal and visceral emotional experiences. The rapid evolution of digital health and oncology informatics has provided a novel framework to address these limitations. Social media platforms such as X (formerly Twitter) function as vast unstructured repositories of longitudinal individual discourse. Natural language processing (NLP) and large-scale data mining, referred to as infodemiology, allow for systematic quantification of patient-reported barriers in a manner that traditional clinical trials cannot easily replicate [3,4].

In the context of breast oncology, barriers to screening are multidimensional and encompass sensory, psychological, economic, and logistical domains. The physical and psychological tolls of mammography are well-documented deterrents. Specifically, in Asian populations, the high prevalence of dense breast tissue necessitates greater compression force, which is directly associated with increased procedural pain [5-7]. Beyond clinical factors, systemic constraints and logistical barriers frequently limit equitable access to preventive health care, adding a significant logistical burden. While the landmark J-START (Japan Strategic Anti-Cancer Randomized Trial) trial established the clinical efficacy of supplemental ultrasound in improving the sensitivity in dense breasts, it did not account for the integrated emotional burden of multimodal screening [8].

A critical yet unexplored informatics question is how these multidimensional barriers, ranging from the sensory salience of procedural discomfort to logistical access, shape the overall screening narrative. Understanding how the highly distressing experience of one modality (mammography) modulates a broader perception of concurrent procedures is a significant structural barrier to screening adherence. Traditional studies have often relied on simple mean sentiment scores, which can obscure cross-cultural differences and emotional volatility. Despite the availability of extensive digital discourse, a rigorous, cross-cultural informatics approach using advanced distributional analysis has not yet been applied to compare the topological structure and affective intensity of screening barriers between Japanese-language and English-language cohorts.

In this study, we leveraged a large language model (LLM)–assisted NLP pipeline executed on a cloud-based informatics platform to analyze over 46,000 screening-related posts in Japanese and English. The objectives were to (1) characterize the divergent topological networks and statistical distributions of multidimensional screening barriers (sensory, psychological, economic, and logistical) across cultures; (2) quantify the temporal transition of sentiment from prescreening anticipation to postscreening reality; and (3) evaluate the impact of mammographic pain on the informatics profile of supplemental ultrasound. By integrating data science with clinical oncology, this study aimed to provide actionable insights to optimize the screening experience and improve global adherence rates.

## Methods

### Data Collection and Preprocessing

Publicly available social media data were retrieved from X (formerly Twitter) using the Apify tweet-scraper-v2, a cloud-based web scraping tool. We collected posts using the following exact Boolean search queries with language filters and chronological sorting (sort: latest): for the Japanese cohort, “(乳癌検診 OR 乳癌健診 OR 乳がん検診 OR 乳がん健診 OR マンモグラフィ OR マンモ OR 乳房エコー OR 乳房超音波 OR 乳腺エコー OR 乳腺超音波) since:2025-01-01 until:2025-12-31 lang:ja”; and for the English-language cohort, “(mammo OR mammogram OR mammography OR “breast ultrasound” OR “breast sonogram” OR “breast cancer screening” OR “breast screening”) since:2025-01-01 until:2025-12-31 lang:en.” No geographic filters or initial exclusion criteria were applied at the scraping stage. To ensure data integrity, a multistep cleaning pipeline was implemented using a LLM Gemini (Flash 3.1-lite; Google AI Studio). After removing exact duplicates and pure reposts (to prevent the artificial amplification of viral posts), we explicitly retained quoted posts and replies, as they contain valuable user-generated sentiments and personal experiences. Subsequently, we used the AI model to systematically identify and exclude automated bot-generated posts, commercial advertisements, spam, irrelevant noise, and news headlines. Specifically, the model was prompted with explicit criteria to flag and remove posts containing promotional URLs, standardized news reporting formats, and unnatural repetitive syntax indicative of bot activity. All subsequent analyses and visualizations were performed exclusively on these final cleaned datasets.

### AI-Based NLP Pipeline and Validation

To overcome the limitations of traditional lexicon-based methods and ensure cross-lingual harmonization, we used an LLM Gemini (Flash 3.1-lite; Google AI Studio) for sentiment scoring and core barrier classification. Instead of relying solely on isolated string matching, we integrated established lexicon metrics as a methodological baseline into the AI’s system prompt. Specifically, the model was calibrated using standardized valence indices ranging from –1.0 (highly negative) to +1.0 (highly positive). This approach aligned with the Semantic Orientations of Words Reference Lexicon [9] for the Japanese cohort and the VADER (Valence Aware Dictionary and Sentiment Reasoner) sentiment suite [10] for the English-language cohort, thereby bridging the linguistic gap.

The AI was systematically prompted to act as an “expert oncology researcher” to analyze the contextual sentiment of each post. To guarantee a standardized evaluation across both languages, we provided explicit scoring heuristics for core screening barriers. These specific heuristic values were clinically calibrated and adjusted by breast oncologists based on the initial lexicon baselines: physical pain or sensory refusal (−0.9), fear or anxiety (−0.7 to −0.8), financial cost (−0.6 to −0.7), and clinical risks (−0.4 to −0.8). Positive sentiments, such as relief, were mapped to a scale of +0.6 to +0.8, with further gradations for recommendations and routine awareness. The system prompt included explicit rules for contextual nuances. For instance, the AI was instructed to correctly handle negations (eg, flipping “no pain” to +0.8) and to strictly map objective factual reports (eg, “Result: Normal” or “Mammogram done”) to a neutral low positive range (0.00 to 0.14) to prevent artificial score inflation. Furthermore, the model was directed to dynamically adjust the intensity of the sentiment scores within the predefined ranges based on modifying adverbs (eg, “very” and “slightly”) and overall contextual severity. To investigate the temporal evolution of screening barriers and the specific impact of screening modalities, the AI was further prompted to act as a medical text annotator. To ensure accurate temporal classification and handle ambiguity, the model was instructed to apply a conservative algorithm. Posts were categorized into 3 temporal phases based on contextual markers: “Before” (anticipatory posts containing intent-based keywords such as “planning to go” or “scared to book”), “After” (experiential posts containing markers such as “just finished” or “was painful”), and “Unknown” (general statements, news quotes, or posts with unspecifiable timing). Any linguistically ambiguous posts lacking clear temporal markers were strictly assigned to the “Unknown” category and excluded from the prescreening and postscreening comparative analysis. Subsequently, based on explicit classification rules, posts were categorized into 5 modalities: “Mammography alone,” “Ultrasound alone,” “Mammography+Ultrasound” (concurrent use of both mammography and ultrasound), “Others” (eg, MRI, palpation), and “Unknown” (modality unspecified). The model systematically extracted the core barriers, continuous sentiment scores, temporal phases, and modalities, returning them in a structured JSON array format to prevent arbitrary text generation and ensure data integrity.

To validate the accuracy of the AI-based NLP pipeline, manual reviews were conducted by independent breast oncologists. For sentiment classification, 200 randomly selected posts (100 per cohort) were independently evaluated by 2 reviewers using a 3-tier classification (positive>+0.1, negative<−0.1, otherwise neutral) to assess interrater reliability and AI-human concordance. Additionally, to validate the temporal and modality classifications, a single independent breast oncologist randomly extracted 100 Japanese posts, manually reviewed the contextual categories, and calculated the concordance rates between the expert’s assessment and the AI’s predictions. Furthermore, to ensure the strict parallelism of the barrier categories and the clinical validity of the cross-lingual translation, the exact correspondence and cultural adaptation of the extracted high-frequency terms between Japanese and English were independently verified by breast oncologists, as detailed in Multimedia Appendix 1.

### Statistical Distribution and Domain-Specific Analysis

Dual-axis lollipop charts were used to integrate keyword frequencies with mean sentiment scores. Sentiment distributions were evaluated using rain cloud plots, which integrate kernel density estimates, jittered raw data, box plots, and ridgeline plots to assess specific screening barrier domains (sensory, psychological, economic, and logistical). To rigorously compare the distributional shapes of continuous sentiment scores across the 4 core barrier domains between the Japanese-language and English-language cohorts, a 2-sample Kolmogorov-Smirnov (KS) test was performed. Differences in sentiment scores between the groups were evaluated using a 2-tailed Welch *t* test, supplemented by the calculation of Cohen *d* and 95% CIs to report effect sizes and precision, addressing the high statistical power inherent in large-scale datasets. All hypothesis tests were 2-sided, and *P*<.001 was considered statistically significant. All analyses and visualizations were performed in Python (version 3.12) with the Pandas, Matplotlib, Seaborn, and SciPy libraries.

### Structural Network Analysis

Co-occurrence network topology analysis was performed using the NetworkX library (Python) to evaluate the interrelationships among the identified screening barriers. Nodes were defined based on the high-frequency core barriers extracted by the NLP pipeline. The co-occurrence window was set at the individual post level (ie, terms co-occurring within the same post). Edge weights were determined by raw co-occurrence frequencies without additional normalization. To filter out sparse connections and optimize visual clarity, the edge threshold was explicitly set to retain the top 40 most frequent co-occurrence pairs within each cohort. This specific threshold was chosen to highlight the primary macrolevel community structures. Furthermore, a sensitivity analysis was conducted by varying the inclusion threshold to include the top 30 and top 50 edges. This analysis confirmed that the fundamental structural topologies of the networks remained highly stable and were not dependent on the specific visualization threshold chosen. Statistical communities within the network were detected using the greedy modularity maximization algorithm.

### Temporal Transition and Modality Impact

To evaluate the temporal evolution of screening barriers, we compared the prevalence and sentiment profiles of the core barriers between the prescreening and postscreening phases. Regarding the impact of modality, we first compared the overall and domain-specific sentiment distributions between posts mentioning mammography (including both standalone and concurrent use) and those categorized as “Ultrasound alone.” Furthermore, to isolate the specific influence of mammography on the perception of ultrasound, we compared the high-frequency barriers and their sentiment profiles between posts categorized as “Mammography+Ultrasound” and those categorized as “Ultrasound alone.”

### Ethical Considerations

This cross-sectional infodemiology study analyzed publicly available social media data, and all data were deidentified. As the study used strictly anonymized and publicly accessible data without any direct interaction with individuals, it was exempt from institutional review board approval and the requirement for informed consent in accordance with local regulatory policies. This study followed the STROBE (Strengthening the Reporting of Observational Studies in Epidemiology) reporting guidelines for cross-sectional studies.

## Results

### Data Collection and Preprocessing

**Figure 1.**
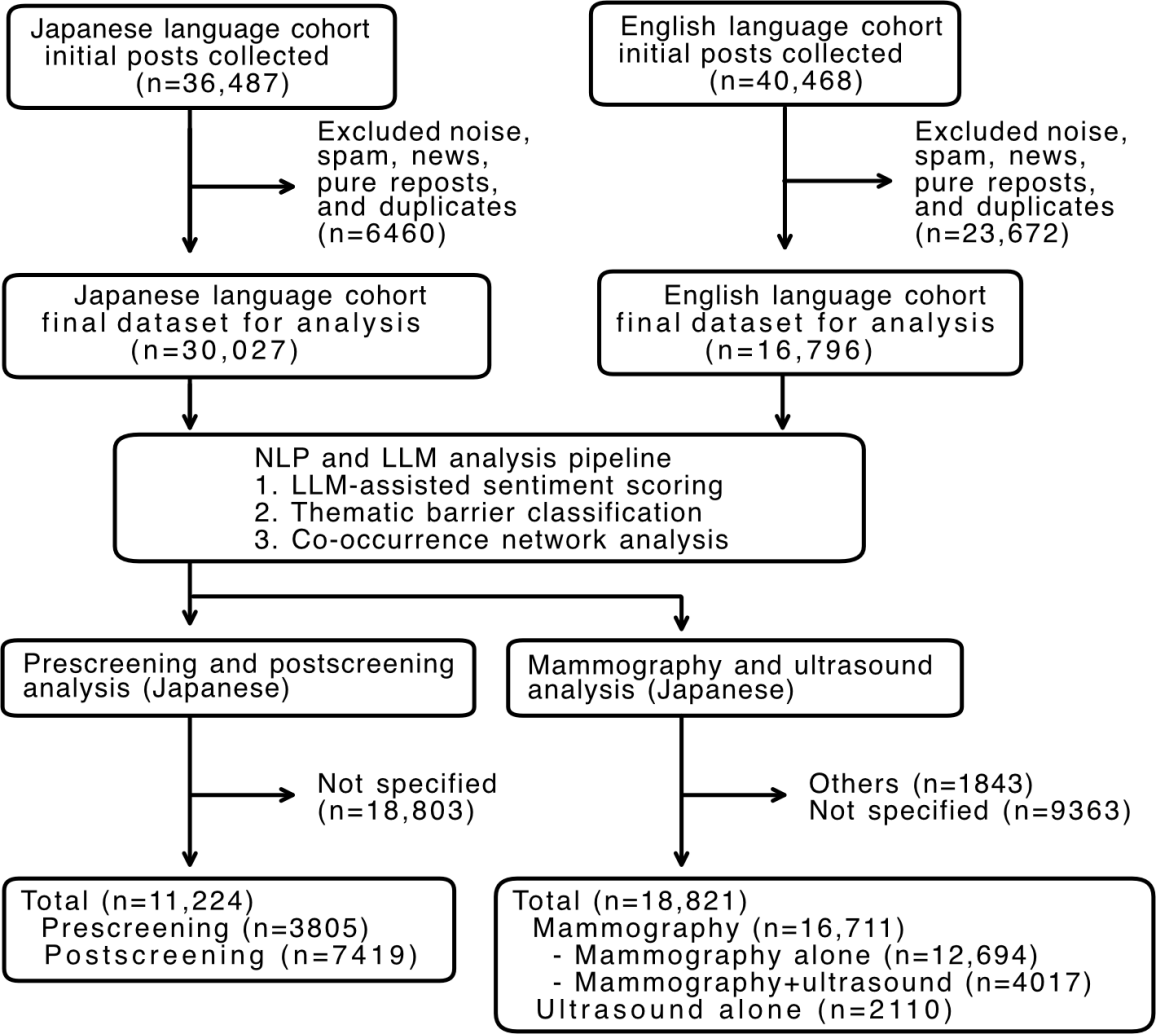
Study selection and data processing flow diagram. The flow diagram illustrates the systematic process of data collection and cleaning for both the Japanese-language and English-language cohorts. Starting with the initial extraction of raw X (formerly Twitter) posts, the diagram details the exclusion of noise, spam, news, pure reposts, and duplicate entries to yield the final datasets. These refined datasets were subsequently processed through an integrated NLP and LLM analysis pipeline, encompassing LLM-assisted sentiment scoring, thematic barrier classification, and co-occurrence network analysis. All subsequent results and figures were generated from these final datasets. LLM: large language model; NLP: natural language processing.

Of the initial 76,955 screening-related posts (36,487 in Japanese and 40,468 in English) generated between January 1 and December 31, 2025, a total of 46,823 posts (30,027 in Japanese and 16,796 in English) were included in the final analysis after excluding 30,132 noise, spam, news, and duplicate entries (6460 in Japanese and 23,672 in English; Figure 1).

### AI-Based NLP Pipeline and Validation

In the manual validation of the 3-tier sentiment classification using 200 randomly selected posts, interrater reliability between 2 independent reviewers was substantial (Japanese: accuracy 84.0%, Cohen κ=0.759; English: accuracy 76.0%, κ=0.641). The AI model demonstrated cross-lingual performance comparable to human experts, achieving accuracies of 79.0%‐91.0% (κ=0.684‐0.860) against individual reviewers in the Japanese-language cohort and 83.0%‐85.0% (κ=0.743‐0.754) in the English-language cohort. Furthermore, for the chronological classification, the overall accuracy across all categories (including “Unknown”) was 76.1% with a Cohen κ of 0.623. This moderate overall agreement was primarily driven by the AI’s conservative algorithm, which categorized linguistically ambiguous posts as “Unknown” to prevent false assignments. When the AI assigned a specific temporal phase, it achieved positive predictive values: 93.2% (68/73) for the postscreening phase and 88.5% (23/26) for the prescreening phase. Cross-phase misclassifications occurred in only 3% (6/200) of cases, supporting the reliability of the prescreening and postscreening datasets used for the downstream temporal analysis. For the screening modality classification, the model achieved an overall accuracy of 94.5% and a Cohen κ of 0.92, demonstrating excellent reliability and high concordance with the expert evaluations.

### Statistical Distribution and Domain-Specific Analysis

**Figure 2.**
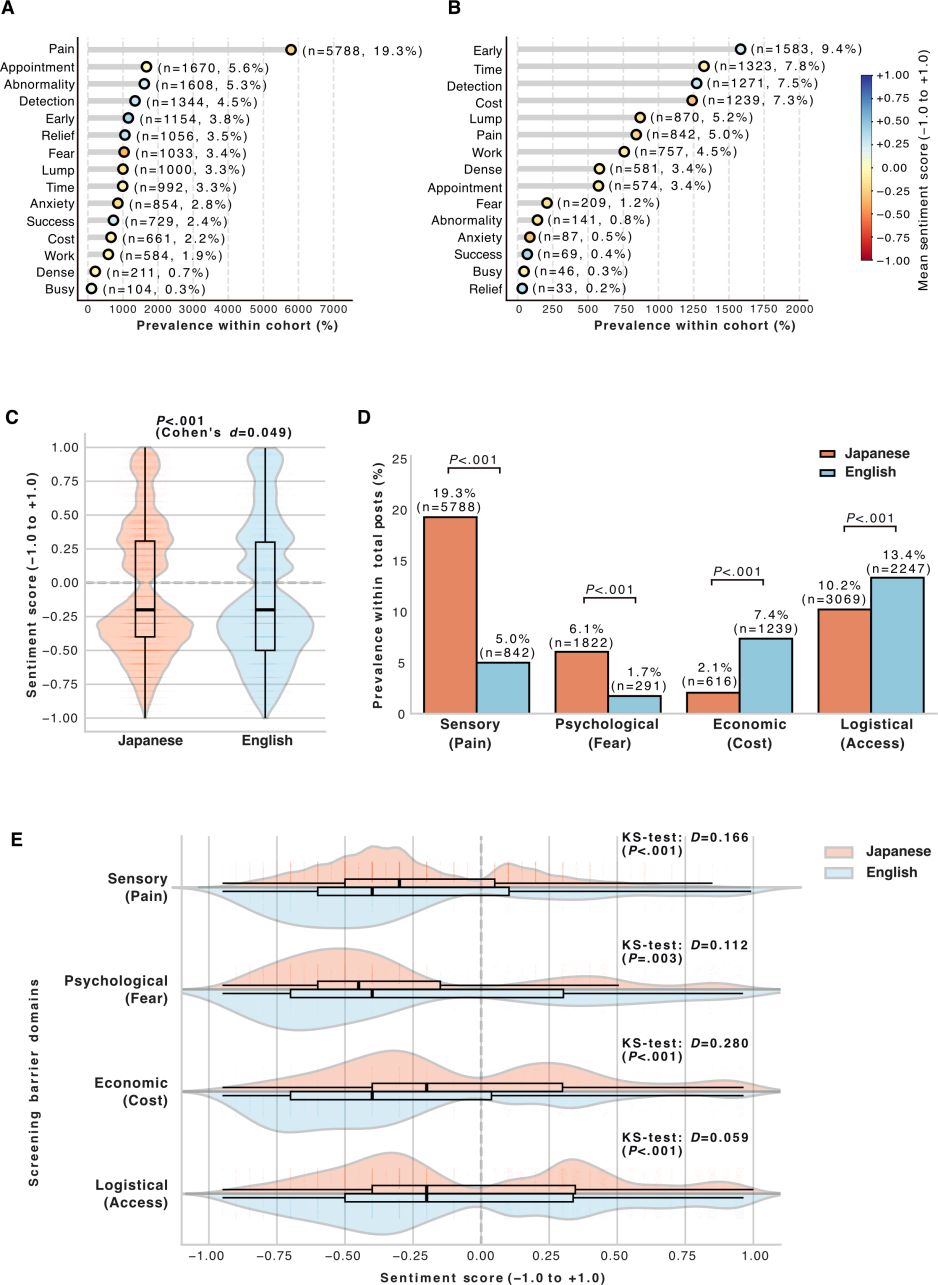
Quantitative landscape of breast cancer screening barriers in Japanese-language and English-language cohorts (A, B) Comparison of the top 15 core barriers and sentiments in breast cancer screening between the Japanese-language and English-language cohorts. The top 15 most frequent terms and their associated sentiment scores are displayed via lollipop charts for (A) the Japanese-language cohort and (B) the English-language cohort. For the lollipop data points, color represents the mean sentiment score, ranging from −1.0 (red, indicating negative sentiment) to +1.0 (blue, indicating positive sentiment), with neutral values shown in white or yellow. The x-axis indicates the frequency (number of posts) and the percentage for each term, while the y-axis lists the core barriers and sentiments. (C) Raincloud plots integrating half-density estimates, jittered raw data points, and box plots to show the distribution of sentiment scores for the Japanese-language (red) and the English-language (blue) cohorts. The sentiment scale ranges from −1.0 (extremely negative) to +1.0 (extremely positive). The thick horizontal bar represents the median, while the box indicates the IQR. A significant difference in sentiment distribution between the 2 cohorts is observed (*P*<.001, 2-tailed Welch *t* test, and Cohen *d*=0.049). (D) Comparison of the prevalence of the 4 domain-specific screening barriers between the Japanese-language and English-language cohorts. Barriers are categorized into 4 predefined dimensions: Sensory (Pain), Psychological (Fear), Economic (Cost), and Logistical (Access). Percentages indicate the proportion of posts containing specific barrier-related terms within each language cohort. Statistical significance was determined using the chi-square test. (E) Ridgeline plots comparing sentiment score distributions across 4 primary screening barrier domains: Sensory (Pain), Psychological (Fear), Economic (Cost), and Logistical (Access). Distributions for the Japanese-language (red) and the English-language (blue) cohorts are overlaid for each domain, with individual data points (ticks) and box-and-whisker plots indicating the median and variance within each category. The x-axis represents the sentiment score from −1.0 (extremely negative) to +1.0 (extremely positive). Statistical significance of the distributional differences was evaluated using the Kolmogorov-Smirnov (KS) test.

Analysis of the top 15 most frequently used terms revealed distinct cross-cultural differences in perceived barriers to and sentiments toward breast cancer screening (Figures 2A and B). The complete correspondence between the extracted Japanese terms and their English translations is provided in Multimedia Appendix 1. Regarding the primary volume of public discourse, the top 3 most dominant topics in the Japanese-language cohort containing 30,027 posts (Figure 2A) were centered on physical sensations, logistics, and clinical anxiety, led by “Pain” (n=5788, 19.3%), followed by “Appointment” (n=1670, 5.6%) and “Abnormality” (n=1608, 5.4%). In contrast, the top 3 most frequent terms in the English-language cohort containing 16,796 posts (Figure 2B) reflected a more proactive, efficacy-driven, and structured discourse, occupied by “Early” (n=1583, 9.4%), “Time” (n=1323, 7.9%), and “Detection” (n=1271, 7.6%).

When isolating true barriers, defined specifically as terms consistently accompanied by marked negative emotional valences (red-to-orange coloration), clear cross-cultural divergences emerged. In the Japanese-language cohort, the primary obstacles were psychosomatic, driven by the anticipation of physical suffering and acute aversions, namely “Pain” and “Fear” (1033/30,027, 3.4%). Conversely, in the English-language cohort, the leading barriers shifted toward socioeconomic and systemic friction rather than physical dread. Financial constraints (“Cost,” 1239/16,796, 7.4%) and the physical discomfort of the procedure (“Pain,” 842/16,796, 5.0%) constituted the top negative-scoring barriers for English-language users. Collectively, these data demonstrate that while the psychological and physical aversions of “Pain” and “Fear” constrain the Japanese-language discourse, the English-language discourse deals primarily with pragmatic, structural obstacles like “Cost” alongside a comparatively muted apprehension toward “Pain.”

At a macroscopic level, the distribution of net sentiment scores showed a similar slightly negative-leaning, widely dispersed emotional profile for both cohorts (Japan: mean −0.059, SD 0.478, 95% CI −0.064 to −0.054; n=30,027; English language: mean −0.082, SD 0.497, 95% CI −0.090 to −0.074; n=16,796; Figure 2C). Although statistically significant (*P*<.001), the clinical or practical difference was negligible (Cohen *d*=0.049), confirming no practically meaningful divergence in the overall emotional equilibrium between the 2 nations. However, deconstructing the discourse into 4 domain-specific barriers revealed sharp cross-cultural disparities in prevalence (Figure 2D). The Japanese-language cohort was constrained by individual physical and emotional aversion, showing significantly higher rates of “Sensory (Pain)” (5788/30,027, 19.3% vs 842/16,796, 5.0%, *P*<.001) and “Psychological (Fear)” barriers (1822/30,027, 6.1% vs 291/16,796, 1.7%, *P*<.001) than the English-language cohort. Conversely, the English-language discourse was predominantly constrained by external and structural frictions, with “Logistical (Access)” (3069/30,027, 13.3% vs 2247/16,796, 10.2%, *P*<.001) and “Economic (Cost)” barriers (616/30,027, 7.4% vs 1239/16,796, 2.1%, *P*<.001) higher than those in the Japanese-language cohort. These findings underscore that while Japanese screening hesitation is largely rooted in somatic anxiety, the friction in the English-language discourse is driven by socioeconomic and systemic limitations.

Finally, we performed the KS test to evaluate the continuous sentiment distribution profiles within each barrier domain, independent of their discussion frequencies (Figure 2E). The “Economic (Cost)” domain exhibited a significant cross-cultural divergence (*D*=0.280, *P*<.001), with the English-language cohort demonstrating a markedly more negative sentiment (mean −0.305, SD 0.474, 95% CI −0.331 to −0.279; n=1237) compared with the Japanese-language cohort (mean −0.058, SD 0.469, 95% CI −0.095 to −0.021; n=615), reflecting that financial barriers trigger a substantially heavier and distinct negative emotional stress in the English-language cohort than in the Japanese-language cohort. Conversely, the distributional differences for the remaining 3 domains were remarkably modest, with small effect sizes observed for “Sensory (Pain)” (*D*=0.166, *P*<.001; Japanese: mean −0.236, SD 0.355, 95% CI −0.245 to −0.227; n=5769 vs English: mean −0.252, SD 0.511, 95% CI −0.287 to −0.217; n=839), “Psychological (Fear)” (*D*=0.112, *P*=.003; Japanese: mean −0.296, SD 0.473, 95% CI −0.318 to −0.274; n=1821 vs English: mean −0.219, SD 0.571, 95% CI −0.285 to −0.153; n=290), and “Logistical (Access)” (*D*=0.059, *P*<.001; Japanese: mean −0.044, SD 0.484, 95% CI −0.061 to −0.027; n=3062 vs English: mean −0.075, SD 0.518, 95% CI −0.096 to −0.054; n=2243). This statistical profile indicates that when these 3 issues are discussed, the underlying emotional intensity and psychological valences remain inherently comparable between the 2 cohorts. Consequently, these statistical profiles suggest that the disparities in the prevalence of “Pain” and “Access” documented in Figure 2D appear to be driven less by cultural differences in emotional sensitivity to these factors and more strongly by the distinct systemic, clinical, and physiological realities of the respective screening environments.

### Structural Network Analysis

**Figure 3.**
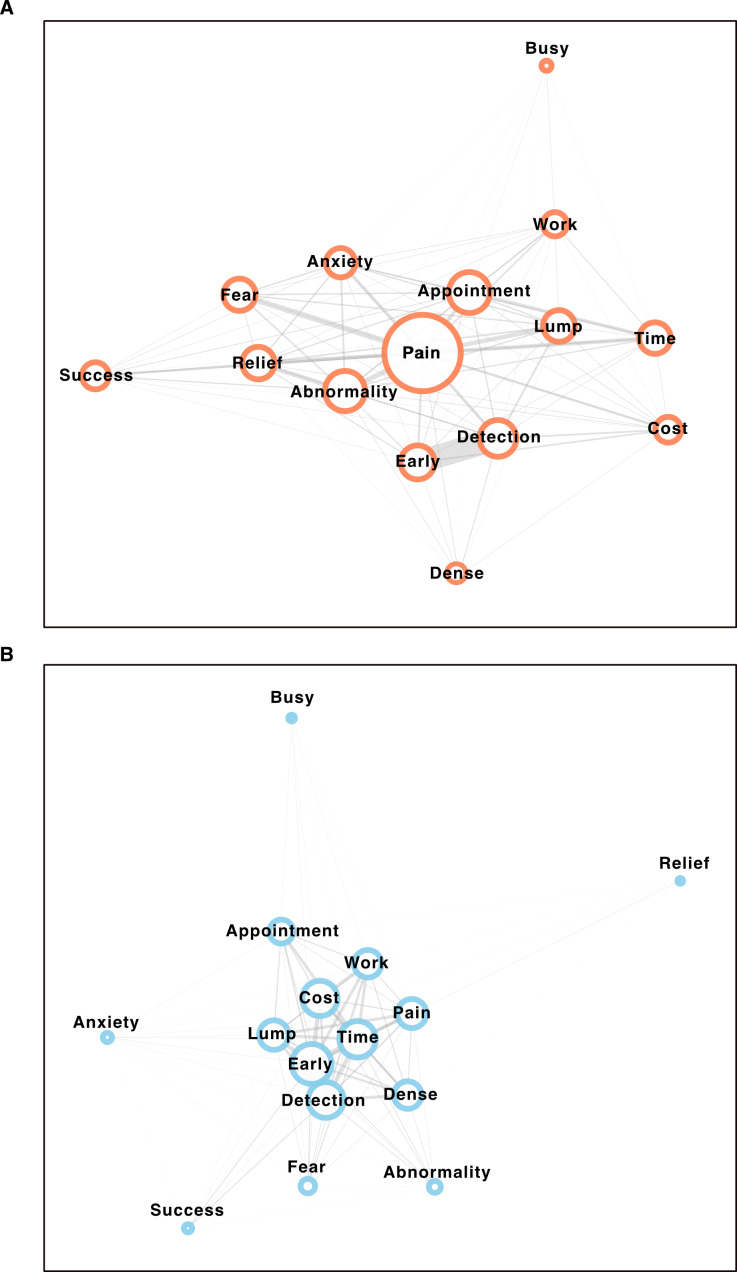
Comparative co-occurrence networks of screening barriers: Japanese-language vs English-language cohorts. (A) Japanese-language cohort (indicated by red nodes). (B) English-language cohort (indicated by blue nodes). Each node represents a high-frequency barrier-related term identified via the LLM-assisted NLP of the social media discourse. Node size is proportional to the frequency of the term within the respective language cohort. The edge width denotes the strength of co-occurrence between concepts within individual posts, with thicker lines indicating more frequent simultaneous mentions. Japanese terms are provided in parentheses to facilitate cross-cultural comparisons.

The co-occurrence network analysis (Figure 3) visually reinforces these structural findings. In the Japanese network, “Pain” emerged as a dominant, high-centrality hub with direct edges to “Fear” and “Appointment.” This topology suggests a tightly coupled narrative where physical aversion (“Pain”) and psychological dread (“Fear”) are linked to the logistical process of scheduling (“Appointment”), creating a complex psychosomatic barrier to screening. In contrast, the English-language network exhibited a more decentralized topology. Rather than converging on a single sensory node, the network featured multiple interconnected hubs, where screening concepts (“Early,” “Detection”) and logistical factors (“Time,” “Cost”) co-occurred broadly with clinical terminology (eg, “Dense”). This configuration indicates that the English-language screening narrative encompasses a diverse range of clinical and systemic topics, whereas the Japanese discourse remains primarily centralized around physical sensation. Additionally, terms reflecting time and occupational constraints (eg, “Work,” “Busy”) demonstrated structural linkages to scheduling logistics (“Appointment”) in both networks, indicating that occupational obligations serve as a shared baseline barrier to screening access.

### Temporal Transition and Modality Impact

**Figure 4.**
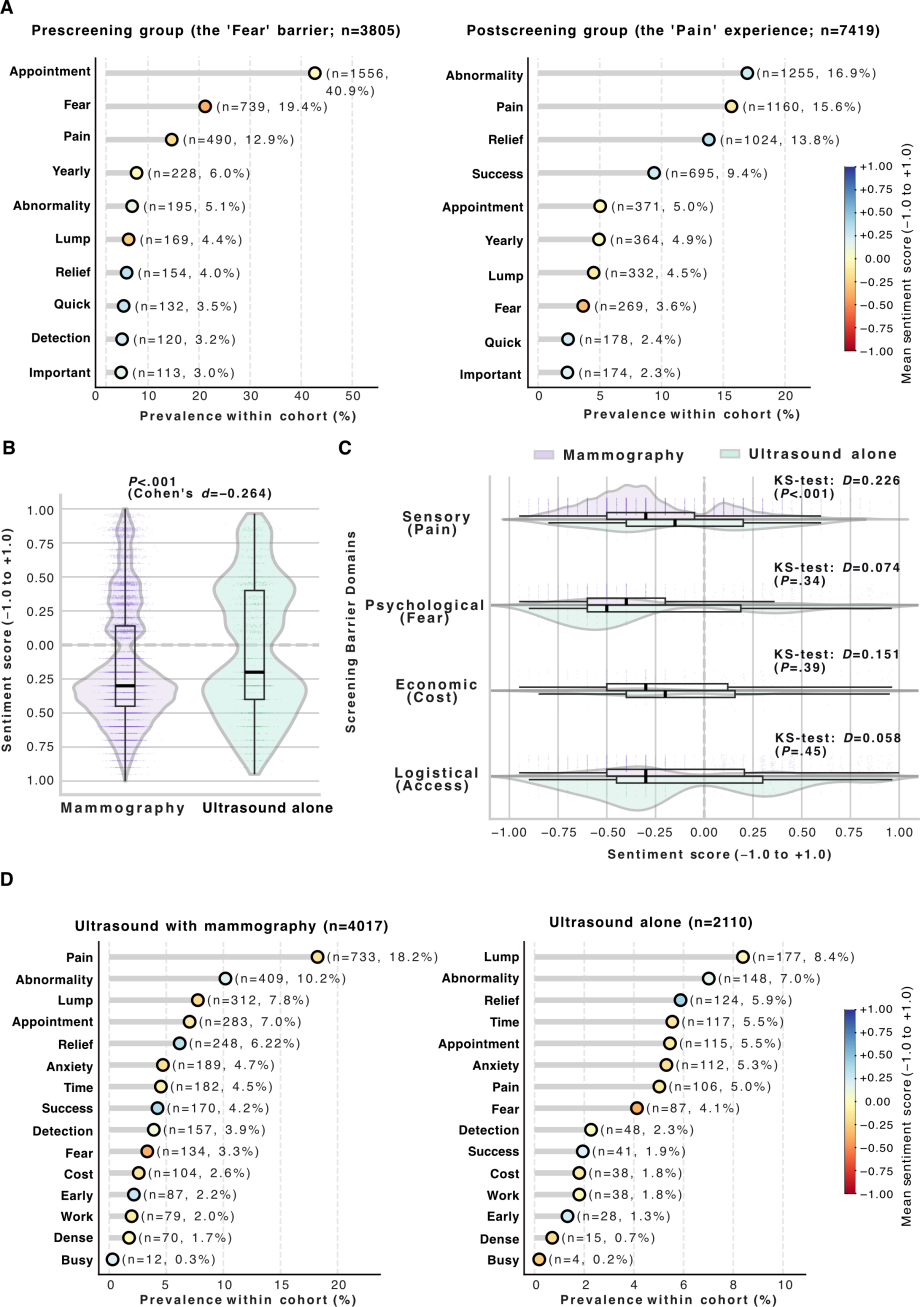
Detailed transition and modality-specific characterization of screening barriers (A) Transition of core barriers and sentiments between prescreening and postscreening groups in the Japanese-language cohort. A comparative analysis of the top 10 terms identified in the prescreening group (n=3805) and the postscreening group (n=7419) is presented. The x-axis represents the prevalence (percentage of posts mentioning the term), and the y-axis lists the key terms identified. Each data point is colored according to its mean sentiment score, ranging from −1.0 (red, negative sentiment) to +1.0 (blue, positive sentiment). (B) Distribution of sentiment scores for mentions of “mammography” (purple) vs “ultrasound alone” without mention of mammography (green). The combined violin and box plots demonstrate a significantly more negative sentiment distribution for mammography compared with ultrasound alone (*P*<.001, 2-tailed Welch *t* test, and Cohen *d*=0.264). (C) Ridgeline plots showing domain-specific sentiment distributions for mammography (purple) and ultrasound alone (green) across sensory, psychological, economic, and logistical barriers. The x-axis represents the sentiment score from −1.0 (extremely negative) to +1.0 (extremely positive), with ticks representing individual data points and box plots indicating medians and IQRs. Statistical significance of the distributional differences was evaluated using the Kolmogorov-Smirnov (KS) test. (D) Impact of mammography co-occurrence on the sentiment profile of ultrasound screening. Comparison of the top 10 terms and their prevalence associated with ultrasound screening between the group mentioning ultrasound alongside mammography (n=4017) and the group mentioning ultrasound alone (n=2110). The x-axis represents prevalence (%), and the y-axis lists the identified terms. Dot colors indicate the mean sentiment score from −1.0 (red) to +1.0 (blue).

To evaluate barrier evolution, Japanese posts were analyzed across the prescreening (n=3805) and postscreening (n=7419) phases, revealing that a significant temporal shift in the thematic focus occurred between the prescreening and postscreening phases (Figure 4A). The prescreening discourse was predominantly driven by logistical hurdles and anticipatory anxiety, with “Appointment” (1556/3805, 40.9%), “Fear” (739/3805, 19.4%), and “Pain” (490/3805, 12.9%) emerging as the top 3 concerns. Following the screening, the discourse transitioned toward outcome-oriented concepts. Result-related terms such as “Abnormality” (1255/7419, 16.9%) and “Relief” (1024/7419, 13.8%) rose to the top echelons, accompanied by strong positive sentiment (blue coloration). Crucially, “Pain” remained prevalent in the postscreening phase as the second most frequent topic (1160/7419, 15.6%). The sustained prominence of “Pain” among the top 3 factors across both time points underscores that physical discomfort is a persistent core element throughout the entire screening continuum, bridging both anticipatory dread and retrospective experience.

A comparison of the overall sentiment scores revealed that posts mentioning mammography (including both stand-alone and combined use; n=16,711) were significantly more negative than those mentioning ultrasound exclusively (n=2110). The mean sentiment score for the mammography group was −0.167 (SD 0.428, 95% CI −0.173 to −0.161) compared with −0.060 (SD 0.459, 95% CI −0.080 to −0.040) for the ultrasound-alone group (*P*<.001). To quantify the magnitude of this difference and address the high statistical power inherent in our large sample size, we calculated the effect size, which indicated a small but statistically significant difference (Cohen *d*=0.264). These findings demonstrate that while the discourse surrounding both modalities tends to be negative, the absence of mammographic compression results in a significantly less negative and relatively more favorable emotional profile.

Across the 4 barrier domains, a significant difference between modalities was observed exclusively in Sensory (Pain). Mammography posts exhibited a significantly more negative sensory sentiment compared to ultrasound-alone posts (mean −0.261, SD 0.350; n=5096 vs mean −0.058, SD 0.404; n=159; *P*<.001; and KS-test *D*=0.226). No significant differences emerged in the psychological (mean −0.312, SD 0.435; n=1005 vs mean −0.257, SD 0.528; n=170), economic (mean −0.146, SD 0.440; n=353 vs mean −0.078, SD 0.484; n=36), or logistical (mean −0.140, SD 0.459; n=1438 vs mean −0.116, SD 0.478; n=255) domains, isolating physical pain as the primary modality-dependent barrier that is mitigated in ultrasound-alone screening.

Finally, to isolate the specific influence of mammography on the perception of ultrasound, the sentiment profiles of ultrasound when mentioned concurrently with mammography (n=4017) and when mentioned separately (n=2110) were compared (Figure 4D). In the concurrent group, “Pain” was the most prominent concern (733/4017, 18.2%). Conversely, in the ultrasound-alone group, “Lump” was the leading term (177/2110, 8.4%), and “Pain” dropped to 5.0% (106/2110). However, the frequencies of psychological barriers remained comparable between the concurrent and ultrasound-alone groups, specifically for “Anxiety” (189/4017, 4.7% vs 112/2110, 5.3%) and “Fear” (134/4017, 3.3% vs 87/2110, 4.1%). These results demonstrate that while the psychological burden of screening remains consistent regardless of modality, the negative pain-related discourse associated with ultrasound is primarily driven by concurrent mammography.

## Discussion

### Principal Findings

This infodemiology study provides a large-scale cross-cultural comparison of breast cancer screening barriers using real-world social media discourse. These findings reveal a structural divergence. While barriers in the English-language cohort are primarily systemic and economic, specifically regarding the financial burden of supplemental screening for dense breasts, the Japanese screening experience is structurally governed by an interconnected network of “Pain,” “Fear,” and “Appointment,” creating a synergistic burden of physical discomfort and logistical access barriers. Domain-specific distributional analyses (KS test) reveal cross-cultural differences. While financial burden (Cost) exerts a distinct emotional stress in English-language discourse, the Japanese screening experience is characterized by emotional volatility. Temporal analyses demonstrate that the Japanese-language cohort transitions from anticipatory anxiety and logistical concerns before screening to physical pain afterward. Finally, modality-specific analyses demonstrate that while the psychological burden of screening remains constant regardless of modality, the physical discomfort of mammography acts as a persistent deterrent that dominates the overall screening experience, overshadowing the benefits of concurrent painless modalities, such as ultrasound.

### Comparison With Prior Work

Patient-reported pain is a well-established predictor of screening nonadherence [11-17]. While previous studies have consistently established that procedural pain and anticipatory fear are primary deterrents to screening adherence, the subgroup analysis expands on this by showing that the discomfort memory of “Pain” persists in the postscreening discourse in Japan, ranking as the second most frequent topic (1160/7419, 15.6%). Crucially, our domain-specific distributional analyses revealed that while the emotional intensity of pain and fear is inherently comparable across cultures, their prevalence is significantly higher in Japan. Furthermore, our network analysis empirically captured that work-related constraints are directly linked to scheduling logistics in both cultures, reflecting known occupational disparities in screening participation [18-20]. In Japan, this baseline logistical burden is structurally compounded by an interconnected network of “Pain,” “Fear,” and “Appointment.” The anticipatory fear of pain is linked to the logistical process of scheduling, transforming the act of booking an appointment into a substantial barrier that drives defensive avoidance [11,21,22].

The significantly higher frequency of pain in the Japanese cohort may be linked to the high prevalence of dense breast tissue [23] and physiological factors. Applying force-standardized protocols, often optimized for Caucasian populations, to the smaller, denser breasts typical of Asian women frequently leads to overcompression and procedural pain [6,7,24]. Furthermore, previous studies have reported that Asians may exhibit lower pain thresholds and tolerance than Caucasians [25], suggesting that inherent differences in sensory sensitivity also contribute to the amplified pain experience. Consequently, the centralized “Pain” hub observed in our Japanese network may manifest from anatomical sensitivity compounded by suboptimal compression protocols. Regarding dense breasts, the structural network reveals a shared cross-cultural understanding rather than a dichotomy, as “Dense” is consistently linked to clinical and physical concepts such as “Detection,” “Abnormality,” and “Pain” in both cohorts. However, there is a distinct cross-cultural disparity in discussion frequency, with “Dense” being discussed more often in the English-language cohort. This elevated public awareness and its prominent presence in the English-language screening narrative are likely driven by US breast density notification laws, which systematically inform women of their density status and its associated risks [26].

A notable contribution of this study is the characterization of the effects of combined modality screening on sentiment. The J-START trial established the clinical efficacy of supplemental ultrasound in dense breasts [8,27,28]; however, these results suggest that this combined approach faces challenges in improving overall adherence. Ultrasound screening alone was associated with less pain than ultrasound screening performed concurrently with mammography. When ultrasonography was performed alongside mammography, pain-related descriptors increased from 5.0% (106/2110) to 18.2% (733/4017). The negative experience of mammography dominates the overall screening narrative, overshadowing the benefits of concurrently performed painless modalities. Mitigating pain-associated avoidance is essential for maximizing the population-level benefits of supplemental ultrasound.

### Limitations

This study had several limitations inherent to infodemiology research. First, data derived from X (formerly Twitter) are subject to inherent platform biases. Users in the Japanese-language and English-language cohorts may differ substantially in age, gender, health literacy, socioeconomic status, and screening history, which restricts the exact generalizability of our findings to the broader screening-eligible populations in each country. Additionally, social media platforms generally suffer from a selection bias that may lead to a possible overrepresentation of highly emotional or negative experiences. Furthermore, the nature of health-related disclosure on social media differs cross-culturally [29-31]. In Japan, the high degree of anonymity on X provides a unique outlet for users to express visceral negative emotions and physical complaints (such as procedural pain) that they might otherwise suppress in formal clinical settings due to cultural norms [32-34]. In contrast, English-language users may use the platform more frequently for health advocacy, information sharing, and discussing systemic or financial burdens. These differences in social media behavior likely amplify both the frequency and emotional intensity of the pain-related discourse in the Japanese-language cohort and the financial discourse in the English-language cohort. Second, despite strict NLP filtering, residual institutional noise may remain, warranting the cautious interpretation of exact quantitative ratios. Third, there are inherent limitations in performing sentiment analysis across different languages. Although we used an LLM and expert validation to harmonize Japanese and English, subtle cross-lingual nuances in emotional expression may still affect the absolute comparability of sentiment scores. Fourth, owing to the anonymized nature of the data, sentiments could not be linked to specific clinical records such as actual breast density, breast size, menstrual cycle timing, or the exact compression force applied. Fifth, because we could not verify unique users, multiple posts may have originated from the same individual. Therefore, the posts are not strictly independent, which could potentially overstate the precision of our reported percentages and statistical tests. Consequently, the reported *P* values and CIs should be interpreted with caution as exploratory and descriptive measures of the data distribution, rather than as strict confirmatory statistical bounds. Sixth, because no geographic filters were applied during data collection, language filters (lang:ja and lang:en) were used to extract the respective discourses. While the Japanese language is highly localized, the English-language cohort undoubtedly includes users from outside the United States. Therefore, it should be noted that the English-language cohort may not strictly represent the entire US population. However, the prominent systemic barriers identified in this cohort (eg, specific out-of-pocket costs and dense breast notification issues) are highly characteristic of US-specific health care policies rather than universal health care systems found in other English-speaking nations. Consequently, in this study, these findings are interpreted as reflecting a broader English-language discourse that is predominantly influenced and shaped by the US screening context. Finally, it is difficult to completely distinguish between posts from patients diagnosed with breast cancer and those from asymptomatic individuals undergoing routine screening. Despite these limitations, our informatics approach successfully overcame the social desirability bias of traditional surveys, providing a large-scale quantification of real-world experiences that drive screening avoidance.

### Conclusions

In this cross-sectional infodemiology study, a fundamental dichotomy emerged: while the English-language discourse predominantly reflects US-specific systemic economic barriers, the Japanese narrative is structurally governed by an interconnected network of “Pain,” “Fear,” and “Appointment.” Distributional analyses have demonstrated that simple mean comparisons obscure cross-cultural differences, particularly the significantly higher prevalence of pain and emotional volatility in Japan. Furthermore, the physical discomfort of mammography acts as a primary deterrent that negatively impacts the overall screening narrative, overshadowing the benefits of concurrent painless modalities such as ultrasound. Improving screening adherence in Japan requires a multifaceted approach: implementing individualized pain-mitigating compression protocols, optimizing clinical workflows to separate mammographic discomfort from supplemental screening to mitigate pain-associated defensive avoidance, and reducing logistical hurdles to improve equitable access.

## Supplementary material

10.2196/97772 Multimedia Appendix 1 Correspondence between English and Japanese terms and extracted sentiments or barriers.

## Abbreviations

J-START: Japan Strategic Anti-Cancer Randomized Trial
KS: Kolmogorov-Smirnov
LLM: large language model
NLP: natural language processing
STROBE: Strengthening the Reporting of Observational Studies in Epidemiology
VADER: Valence Aware Dictionary and Sentiment Reasoner
